# The Urogenital Microbiome–Metabolic Interface in Postmenopausal Recurrent Urinary Tract Infection: Estrogen Deficiency, Diabetes, Obesity, and Microbial Reservoirs

**DOI:** 10.3390/jcm15155895

**Published:** 2026-07-28

**Authors:** Wasim Iyad Alghoul, Reem Ashraf, Samreen Rafiuddin, Ahmad Ziad Hasan Kharoufeh, Wed Burhan Jameel Al-Shammari, Malak Abedi, Ibrahim Alabid, Mohamedanas Mohamedfaruk Patni, Ali Jad Yousef, Hesham Amin Hamdy

**Affiliations:** 1 RAK College of Medical Sciences, Ras Al Khaimah Medical and Health Sciences University (RAKMHSU), Ras Al Khaimah P.O. Box 11172, United Arab Emirates; wasim.21901111@rakmhsu.ac.ae (W.I.A.); reemoozoozoo@gmail.com (R.A.); samreenrafiuddin2@gmail.com (S.R.); ahmadkiswani51@gmail.com (A.Z.H.K.); wed.23901093@rakmhsu.ac.ae (W.B.J.A.-S.); malak.abedi0320@gmail.com (M.A.); 2 Department of Community Medicine, RAK College of Medical Sciences, Ras Al Khaimah Medical and Health Sciences University (RAKMHSU), Ras Al Khaimah P.O. Box 11172, United Arab Emirates; 3 Department of Surgery, RAK College of Medical Sciences, Ras Al Khaimah Medical and Health Sciences University (RAKMHSU), Ras Al Khaimah P.O. Box 11172, United Arab Emirates; alijad@rakmhsu.ac.ae (A.J.Y.); hesham@rakmhsu.ac.ae (H.A.H.)

**Keywords:** postmenopause, recurrent urinary tract infection, urogenital microbiome, vaginal microbiome, urinary microbiome, estrogen deficiency, diabetes mellitus, obesity

## Abstract

**Background/Objectives:** Recurrent urinary tract infection (rUTI) is common after menopause, but estrogen deficiency alone does not explain variation in recurrence, symptoms, and microbial profiles. This study aimed to synthesize evidence on the interactions among estrogen deficiency, urogenital microbial ecology, diabetes, obesity, microbial reservoirs, and anatomical, neurological, and functional modifiers of postmenopausal rUTI susceptibility. **Methods:** PubMed and Scopus were searched for original studies published from 1 January 2021 to 15 June 2026. A structured narrative synthesis included 62 original reports comprising clinical interventions, observational cohorts, microbiome and multi-omic studies, and cellular and animal experiments. Evidence was interpreted according to study design, population relevance, and biological directness. **Results:** Menopause was associated with reduced *Lactobacillus* dominance, higher vaginal pH, and altered vaginal or urinary communities, although findings were heterogeneous. Vaginal estrogen reduced recurrence and improved the local urogenital environment, but microbiome restoration is not established as its sole mechanism. Gut, rectal, vaginal, urinary, and bladder-wall reservoirs may contribute to persistence or repeated exposure. Diabetes was linked to dysbiosis and impaired urothelial defence, whereas obesity evidence remained less direct. Pelvic organ prolapse with incomplete emptying, elevated postvoid residual urine, age-related detrusor dysfunction, stroke, immobility, functional dependence, incontinence, and catheter exposure may further modify susceptibility. **Conclusions:** Postmenopausal rUTI is multifactorial. The urogenital microbiome–metabolic interface is a useful integrative framework, but not a validated causal or diagnostic model. Vaginal estrogen, urine culture, metabolic and bladder-function assessment, and antimicrobial stewardship remain the clinical foundation; microbiome-directed strategies require prospective validation.

## 1. Introduction

Recurrent urinary tract infection (rUTI) is generally defined as at least two symptomatic episodes within six months or at least three within twelve months, although requirements for microbiological confirmation vary across studies [1,2]. Recurrence exposes patients to repeated diagnostic testing and antimicrobial treatment, including prolonged prophylaxis, while increasing the likelihood of resistant organisms and antibiotic-recalcitrant disease [1,3,4]. The burden is particularly important after menopause, when lower urinary tract changes, hypoestrogenism, incontinence, incomplete emptying, and comorbidity may coexist [3,4,5]. Yet recurrence is not uniform among older women, indicating that age or estrogen loss alone cannot fully explain individual susceptibility.

The traditional model of a sterile urinary tract has been replaced by evidence that viable bacteria and bacterial DNA can be detected in urine from women without conventional culture-confirmed infection. Expanded quantitative urine culture and sequencing studies have identified distinct low-biomass urinary communities in asymptomatic women and those with recurrent cystitis [1,6,7,8]. This ecological perspective complicates the distinction between commensal colonization, dysbiosis, asymptomatic bacteriuria, and symptomatic infection. It also highlights the importance of sampling and analytical method: standard culture may miss clinically relevant organisms, whereas voided specimens may incorporate urethral or vulvovaginal microbiota, and sequencing cannot by itself establish microbial viability or pathogenicity [2,9].

Menopause substantially remodels the urogenital environment. Across longitudinal and cross-sectional cohorts, postmenopausal status has been associated with less frequent *Lactobacillus*-dominant vaginal communities, greater representation of diverse anaerobic or opportunistic taxa, and higher vaginal pH [6,10,11,12]. These changes are consistent with reduced epithelial estrogen stimulation and diminished support for lactic-acid-producing organisms. However, microbiome composition varies among postmenopausal women, and associations with vaginal atrophy, urinary symptoms, or genitourinary syndrome of menopause are not uniform [9,10,11,13]. Estrogen deficiency therefore appears to create a susceptible ecological state rather than determine a single postmenopausal microbial phenotype.

Susceptibility may be further modified by microbial reservoirs and metabolic disease. Multi-compartment and longitudinal studies support contributions from intestinal, rectal, vaginal, urinary, and bladder-wall bacterial populations, although carriage does not invariably progress to symptomatic recurrence [14,15,16,17]. Diabetes is associated with altered vaginal and urinary microbial composition and impaired urothelial defence; mechanistic studies demonstrate effects of hyperglycaemia and reduced insulin-receptor signalling on epithelial barrier integrity and antimicrobial peptide expression [18,19,20,21]. Obesity has also been associated with reduced vaginal *Lactobacillus* dominance and increased local inflammation, while experimental work links diet-induced obesity to enhanced uropathogenic *Escherichia coli* invasion through altered bladder signalling [22,23,24]. The obesity evidence, however, remains less directly applicable to postmenopausal rUTI than the evidence for estrogen deficiency or diabetes.

Most studies examine these hormonal, microbial, immunological, and metabolic components separately. This fragmentation limits interpretation of why some postmenopausal women develop repeated infection while others with comparable estrogen deficiency remain asymptomatic. Accordingly, this review integrates evidence for an estrogen–microbiome–metabolic interface in postmenopausal rUTI, distinguishes direct clinical findings from indirect or experimental support, and identifies the translational gaps that must be resolved before microbiome-informed risk stratification or treatment can enter practice.

## 2. Literature Search Strategy and Study Selection

This structured narrative review was designed to synthesize original evidence concerning the interaction among menopause, estrogen deficiency, urogenital microbial ecology, recurrent urinary tract infection, diabetes, obesity, antimicrobial exposure, host defence, and microbial reservoirs. PubMed and Scopus were searched for studies published from 1 January 2021 to 15 June 2026. Complete database-specific PubMed and Scopus search strings, including field restrictions, publication-date limits, document-type filters, and language restrictions, are provided in Supplementary File S1.

The search strategy was built using Boolean operators and combined four major concept blocks. The first block captured recurrent infection using: (“recurrent urinary tract infection” OR “recurrent urinary tract infections” OR “recurrent UTI” OR rUTI OR “recurrent cystitis” OR recurrent AND [“urinary tract infection” OR UTI OR cystitis]). The second block captured sex, menopausal, and genitourinary-syndrome context using: (women OR female OR postmenopaus* OR menopause OR menopaus* OR perimenopaus* OR “genitourinary syndrome of menopause” OR GSM). The third block captured microbiome, reservoir, omics, and resistance concepts using: (microbiome OR microbiota OR urobiome OR “urinary microbiome” OR “vaginal microbiome” OR “urogenital microbiome” OR “gut microbiome” OR dysbiosis OR Lactobacillus OR metagenomic* OR metabolomic* OR “16S” OR reservoir* OR rectal OR intestinal OR gut OR “bladder wall” OR bladder-wall OR intracellular OR strain OR “antimicrobial resistance” OR resistome OR “expanded quantitative urine culture” OR EQUC). The fourth block captured hormonal, metabolic, diagnostic, therapeutic, and antimicrobial modifiers using: (estrogen OR oestrogen OR estradiol OR estriol OR diabetes OR “type 2 diabetes” OR hyperglyc* OR SGLT2 OR obesity OR obese OR adiposity OR “body mass index” OR BMI OR antibiotic* OR antimicrobial* OR prevention OR diagnosis OR treatment OR recurrence).

**Figure 1:**
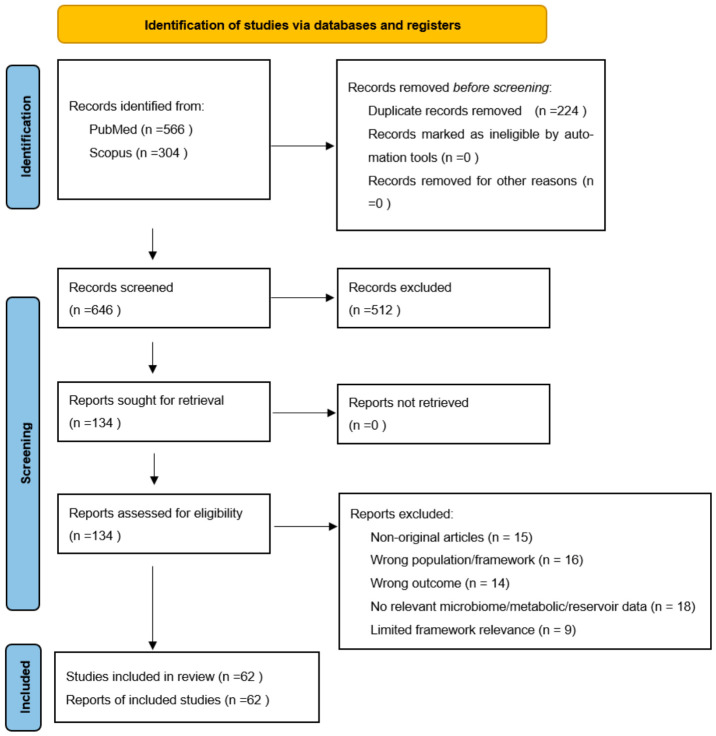
Modified study-selection flow diagram. The diagram summarizes database identification, duplicate removal, title and abstract screening, full-text eligibility assessment, exclusion reasons, and final inclusion of 62 original reports in the narrative synthesis.

The searches identified 870 records: 566 from PubMed and 304 from Scopus. After removal of 224 duplicate records, 646 unique records were screened by title and abstract, of which 512 were excluded. A total of 134 reports were sought for retrieval, and all were obtained and assessed for eligibility. After full-text assessment, 72 reports were excluded: non-original article type (n = 15); wrong population or framework (n = 16); wrong outcome (n = 14); absence of relevant microbiome, metabolic, reservoir, host-defence, anatomical, neurological, or functional evidence (n = 18); or limited relevance to the review framework (n = 9). The final narrative synthesis included 62 eligible original reports. The study-selection process is summarized in Figure 1.

Eligible publications included randomized or controlled interventions, prospective and retrospective cohorts, cross-sectional microbiome studies, longitudinal multi-omic investigations, diagnostic or culture-method studies, and translational animal or cellular experiments directly relevant to urinary infection susceptibility, microbial reservoirs, antimicrobial resistance, or host defence. Reviews, editorials, protocols, conference abstracts, non-original articles, studies outside the publication window, and studies without meaningful relevance to the hormonal, microbial, metabolic, reservoir, diagnostic, therapeutic, or recurrent-UTI components were excluded from the final synthesis.

For interpretation, studies were categorized as direct postmenopausal rUTI evidence; postmenopausal microbiome or genitourinary-syndrome evidence; broader female rUTI microbiome or reservoir evidence; diabetes-associated evidence; obesity-associated evidence; therapeutic evidence; diagnostic evidence; or experimental mechanistic evidence. Human clinical evidence was prioritized over indirect epidemiological, animal, cellular, or exploratory omics findings. Publications derived from overlapping populations or secondary analyses were retained when they addressed distinct outcomes but were treated as related analyses rather than independent cohorts. Evidence strength was interpreted according to study design, population relevance, longitudinality, biological directness, consistency across studies, and whether the findings were clinical, translational, experimental, or hypothesis-generating.

## 3. The Postmenopausal Urogenital Ecosystem

### 3.1. Microbial Communities of the Female Urinary and Vaginal Tracts

The female urinary tract is no longer regarded as uniformly sterile. Enhanced culture and molecular sequencing have detected low-biomass microbial communities in women without conventional culture-confirmed infection, with *Lactobacillus*, *Gardnerella*, *Prevotella*, *Streptococcus*, and other genera recurring across urinary profiles [1,2,6,8]. These findings have shifted interpretation from a binary sterile-versus-infected model toward an ecological framework in which microbial detection, community structure, host response, and symptoms must be considered together. Nevertheless, the identification of bacterial DNA or cultivable organisms does not establish that a detected taxon is pathogenic. Colonization, contamination, asymptomatic bacteriuria, and symptomatic infection remain biologically and clinically distinct states.

The vaginal and urinary microbiota are anatomically related but are not interchangeable. Vaginal communities are more frequently dominated by *Lactobacillus*, whereas urine contains a lower-biomass and often more heterogeneous community. Studies sampling both compartments have demonstrated shared taxa but also clear niche-specific patterns, including stronger vaginal *Lactobacillus* dominance and a greater urinary representation of potential uropathogens [25,26]. Consequently, organisms detected in voided urine may originate from the bladder, urethra, vulva, vagina, or perineum, whereas catheterized specimens more closely approximate the bladder environment.

### 3.2. Microbial Remodelling Across Menopause

Menopause is consistently associated with restructuring of the vaginal and urinary microbiota. Premenopausal urine is more commonly characterized by *Lactobacillus*-dominant urotypes, whereas postmenopausal urine shows greater diversity and increased representation of genera such as *Gardnerella* and *Prevotella* [6]. Vaginal studies similarly demonstrate reduced *Lactobacillus* dominance, increased vaginal pH, and a shift toward diverse anaerobic or opportunistic communities after menopause [10,11,12,27]. Species-level patterns may also differ: some studies identified greater representation of *Lactobacillus crispatus* before menopause and *Lactobacillus iners* after menopause, underscoring that the presence of the genus alone may not fully describe ecological function [25].

These changes are heterogeneous rather than universal. A substantial proportion of postmenopausal women retain *Lactobacillus*-rich communities, while others exhibit stable, highly diverse profiles [10,28]. Longitudinal evidence suggests that low-*Lactobacillus* states may persist over time rather than representing transient disturbances. Menopausal status may therefore be a stronger determinant of microbial composition than rUTI history in some populations, but it does not define a single postmenopausal microbiome [25,29].

### 3.3. Relationship with GSM and Urinary Symptoms

Associations between microbial composition and genitourinary syndrome of menopause are inconsistent. *Lactobacillus*-dominated communities have been associated with lower odds of vaginal dryness, atrophic changes, and some sexual symptoms, whereas diverse communities containing *Gardnerella*, *Escherichia–Shigella*, *Anaerococcus*, *Enterococcus*, or related taxa have been linked to greater symptom burden or local inflammation [10,13,29,30]. However, effect sizes are often modest, and some studies have found limited or no association between *Lactobacillus* dominance and urinary symptoms. In a urinary microbiome study of postmenopausal women with GSM, overall diversity did not differ significantly according to urinary symptom status, although *Prevotella* was enriched among symptomatic participants [28].

These findings indicate that hypoestrogenism and dysbiosis do not translate uniformly into symptoms. Urinary complaints may relate more closely to bladder microbial communities, epithelial integrity, neuromuscular function, or comorbid disease than to vaginal composition alone. The postmenopausal urogenital ecosystem should therefore be understood as a susceptibility state shaped by hormonal, microbial, anatomical, and host factors rather than as a deterministic microbial disorder.

### 3.4. Methodological Context

Interpretation of urogenital microbiome studies is strongly influenced by methodology. Standard urine culture favors rapidly growing conventional uropathogens and may fail to detect organisms recovered by expanded quantitative culture [2]. Voided urine is more vulnerable to periurethral, vulvar, and vaginal contribution than catheterized urine, whereas catheterized specimens more closely approximate bladder-associated communities but are more invasive. These differences are particularly important in low-biomass urinary samples, where small differences in collection, storage, extraction, amplification, and contamination control can substantially affect results.

Sequencing-based studies also have important limitations. Partial 16S rRNA sequencing provides useful genus-level community information but may inadequately distinguish species with different biological roles, whereas full-length 16S sequencing and shotgun metagenomics offer greater taxonomic or functional resolution at higher cost and complexity [13,27]. Sequencing detects microbial DNA and therefore cannot, by itself, distinguish viable organisms from dead bacteria, extracellular DNA, transient contamination, or clinically meaningful colonization. Batch effects, reagent contamination, differences in DNA-extraction methods, primer selection, sequencing platforms, reference databases, and bioinformatic pipelines may all influence the taxa detected and their relative abundance.

Relative-abundance data create additional interpretive challenges because an apparent increase in one taxon may reflect a decrease in another rather than a true increase in absolute microbial load. Many studies also lack absolute quantification, standardized negative controls, strain-level resolution, longitudinal sampling, or paired host-response measurements. Consequently, microbial detection should not be equated with infection, pathogenicity, or therapeutic targetability. These methodological limitations help explain why studies do not consistently identify one protective postmenopausal community or one universal microbial signature of rUTI.

The included studies varied considerably in population, anatomical sampling site, analytical platform, and proximity to clinically confirmed postmenopausal rUTI. Table 1 summarizes ten representative studies selected to illustrate the principal hormonal, microbial, metabolic, interventional, reservoir, and mechanistic evidence domains considered in this review.

## 4. Biological Drivers of Recurrent UTI Susceptibility

The following sections distinguish direct human clinical evidence from translational, experimental, and hypothesis-generating findings. Evidence from randomized or controlled clinical studies and human cohorts is prioritized when available, whereas animal experiments, cellular studies, and indirect mechanistic observations are interpreted as biologically plausible but not clinically proven. Accordingly, the proposed urogenital microbiome–metabolic interface is presented as an evidence-informed susceptibility framework rather than as an established causal pathway.

### 4.1. Estrogen Deficiency, Epithelial Ecology, and Immune Regulation

Estrogen deficiency modifies the postmenopausal urogenital environment through linked epithelial, biochemical, and microbial changes. Reduced estrogenic stimulation is associated with epithelial thinning, diminished maturation, lower glycogen availability, and reduced support for lactic-acid-producing organisms. The resulting rise in vaginal pH may weaken colonization resistance and facilitate expansion of anaerobic or opportunistic taxa. Human studies support this sequence at the ecological level: urinary estrogen metabolites correlate positively with *Lactobacillus* and *Bifidobacterium*, and estrogen exposure is associated with enrichment of these genera in postmenopausal urogenital communities [31,34]. Nevertheless, estrogen deficiency does not produce a uniform microbial state, and individual responses depend on baseline community structure, treatment exposure, and anatomical site.

The functional relevance of estrogen extends beyond community composition. In postmenopausal women with rUTI, vaginal estrogen was associated with reduced urothelial shedding, lower urinary inflammatory scores, and decreased urinary interleukin-6, suggesting modulation of local inflammatory activity [3]. In a separate randomized-trial analysis, vaginal estradiol produced substantial changes in vaginal metabolites, including increased lactate, alongside lower pH and greater dominance by *Lactobacillus* or *Bifidobacterium* [34]. By contrast, a urinary microbiome study found reduced *Finegoldia magna* after local estrogen without a corresponding rise in urinary *Lactobacillus*, indicating that estrogen-related protection may involve suppression of selected organisms, epithelial effects, or immune modulation rather than a single reproducible taxonomic shift [35].

Experimental evidence further links estrogen to urothelial defence under metabolic stress. High glucose reduced psoriasin and other antimicrobial mediators, impaired epithelial barrier properties, and increased *Escherichia coli* burden; estradiol treatment partially restored cellular function and bacterial killing in uroepithelial models [18]. These findings provide a mechanistic bridge between hypoestrogenism and diabetes, but they do not establish that estrogen prevents rUTI primarily through psoriasin in clinical populations. Overall, the evidence supports estrogen as a regulator of epithelial ecology and host defence while arguing against reduction in its effect to restoration of *Lactobacillus* alone. Thus, the human intervention evidence supports a clinical benefit of vaginal estrogen, whereas the specific mechanisms linking estrogen, antimicrobial peptides, epithelial defence, and microbiome restoration remain incompletely established and partly hypothesis-generating.

### 4.2. Microbial Reservoirs, Transmission, and Persistence

Recurrent infection may arise through reinfection from extra-urinary reservoirs, persistence within the urinary tract, or both. Vaginal and urinary profiling studies show that women with rUTI differ from healthy controls or women with sporadic cystitis in community composition, but the reported taxa are inconsistent. Alterations have included reduced protective lactobacilli and enrichment of Enterobacterales, *Escherichia–Shigella*, *Prevotella*, *Enterococcus*, *Streptococcus*, *Anaerococcus*, and other organisms [7,8,36,37]. This heterogeneity suggests that rUTI is not defined by a single dysbiotic configuration. Species-level composition, functional capacity, antibiotic exposure, and host context may be more informative than alpha diversity alone.

The intestine is a plausible source of repeated uropathogen exposure. In a year-long study, women with a history of rUTI had lower gut microbial richness and reduced abundance of butyrate-producing bacteria, while gut and bladder *E. coli* abundance and phylogroup distributions were broadly comparable between groups. The same cohort demonstrated systemic immune differences, supporting a model in which gut dysbiosis may modify susceptibility through immune function as well as pathogen carriage [17]. A larger multicentre cohort did not identify broad gut-community differences between patients with and without recurrence, but asymptomatic urinary colonization was associated with higher gut *E. coli* abundance and greater antimicrobial resistance [16]. These results indicate that reservoir biology may depend on strain persistence and host–microbiome interactions rather than total *E. coli* abundance alone.

Other studies provide complementary but not uniformly concordant evidence. Gut dysbiosis was associated with recurrent rather than sporadic UTI in one female cohort, with increased body weight also associated with dysbiosis [38]. Conversely, no gut microbiome characteristic independently predicted subsequent UTI among long-term aged-care residents after adjustment for age-related and clinical factors [4]. This negative finding is informative because antibiotic exposure, frailty, incontinence, diabetes, and marked interindividual variability may obscure associations detectable in younger or less medically complex populations.

Evidence from simultaneous sampling strengthens the reservoir concept. Perimenopausal women with recurrent vaginitis and UTI showed differences across urinary, cervical, vaginal, anal, and oral sites, with the largest functional alterations identified in anal and urinary communities [14]. In postmenopausal women, paired rectal cultures and vaginal sequencing demonstrated frequent carriage of antibiotic-resistant uropathogens and concordance across rectal, vaginal, and urinary compartments [15]. Such concordance supports a gut–perineal–vaginal–bladder pathway, but cross-sectional similarity cannot prove the direction or timing of transmission. Longitudinal strain-resolved sampling is required to distinguish repeated seeding from parallel colonization.

Persistence within the bladder provides an additional explanation for antibiotic-recalcitrant disease. Among women undergoing electrofulguration of chronic cystitis lesions, 73% of those with positive cultures before and after the procedure grew the same organism, most commonly *E. coli* [39]. In bladder biopsies from 34 menopausal women, tissue-embedded bacteria were detected by fluorescence in situ hybridization, and a high total bladder-wall bacterial burden was associated with earlier relapse after electrofulguration, with a hazard ratio of 3.15, although *Escherichia*-specific burden was not independently significant [40]. These observations support tissue persistence as a clinically relevant mechanism in selected refractory cases but do not establish electrofulguration as a microbiome-directed treatment for routine rUTI.

**Figure 2:**
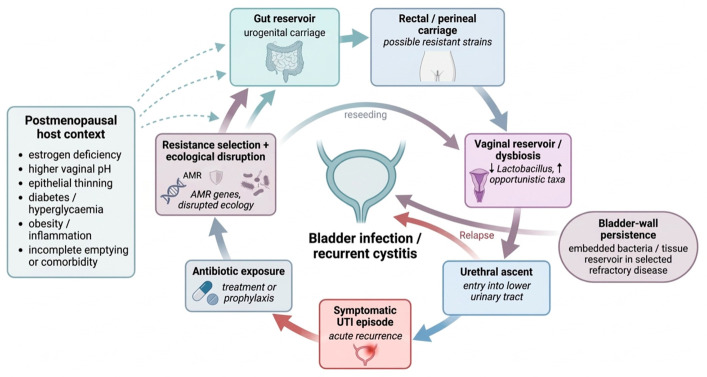
Reservoir–recurrence cycle in postmenopausal recurrent urinary tract infection. Recurrent UTI may arise from repeated uropathogen exposure from gut, rectal, perineal, and vaginal reservoirs, with urethral ascent into the bladder. In selected refractory cases, bladder-wall persistence may contribute to relapse. Postmenopausal estrogen deficiency, diabetes, obesity-related inflammation, incomplete emptying, antimicrobial exposure, and AMR may facilitate recurrence. This model represents a proposed evidence-informed cycle rather than a validated universal causal pathway. Abbreviations: AMR, antimicrobial resistance; rUTI, recurrent urinary tract infection; UTI, urinary tract infection. Created in BioRender. Alabid, I. (2026) https://BioRender.com/30fxa4t.

Metagenomic analysis has shown enrichment of antimicrobial-resistance genes in postmenopausal urogenital communities with an rUTI history, including when no active infection was present [31]. A large hospital cohort similarly documented substantial multidrug resistance among postmenopausal women with symptomatic UTI [4]. Repeated antimicrobial exposure may connect reservoir persistence with ecological disruption. Antibiotics may select resistant organisms and reduce colonization resistance, while recurrent disease itself drives repeated antibiotic use. Both processes may reinforce one another. The proposed reservoir–recurrence cycle is illustrated in Figure 2.

### 4.3. Diabetes: Microbial Alteration and Potential Impairment of Urothelial Defence

The diabetes evidence includes human observational microbiome studies, large clinical risk-factor cohorts, and mechanistic animal or cellular studies. These evidence types support biological plausibility but should not be interpreted as proving that diabetes-associated dysbiosis directly mediates postmenopausal rUTI.

Diabetes may increase rUTI susceptibility through simultaneous effects on microbial ecology and host defence. Urinary microbiome studies in type 2 diabetes have reported higher total bacterial burden, increased Bacillota, and qualitative species-level differences compared with metabolically healthy controls, with some changes most apparent in women [41]. Vaginal studies similarly show reduced *Lactobacillus*, increased diversity, and greater representation of anaerobic or opportunistic communities in women with diabetes [19,20]. In peri- and postmenopausal participants, *Lactobacillus* abundance was markedly lower in diabetes, while fasting glucose and HbA1c correlated negatively with *L. crispatus*, *L. gasseri*, and *L. iners* [19]. These associations link metabolic control with microbial composition but do not establish that dysbiosis mediates clinical recurrence.

Hyperglycaemia also weakens urothelial innate immunity. Human urinary cells, clamp experiments, diabetic mice, and uroepithelial cell models showed that elevated glucose reduced psoriasin expression, impaired cytokine signalling and barrier function, and increased *E. coli* burden [18]. The effect was related to glucose rather than hyperinsulinaemia alone. This supports a mechanism in which poor glycaemic control increases vulnerability through impaired antimicrobial activity, in addition to providing urinary glucose that may favour bacterial growth.

Insulin resistance may contribute independently of severe hyperglycaemia. In diabetic mouse models, reduced urothelial insulin-receptor signalling increased UTI susceptibility, whereas receptor activation was protective. Urothelial insulin-receptor deletion disrupted barrier genes, suppressed antimicrobial peptides, and attenuated nuclear factor-κB activation; similar reductions in insulin-receptor, barrier, and antimicrobial transcripts were identified in urinary cells from young people with type 2 diabetes [21]. Because much of this evidence is experimental and the human samples were not postmenopausal, the pathway should be interpreted as mechanistic support rather than direct proof in the target population.

The relationship between sodium–glucose cotransporter-2 inhibitors and bacterial UTI is more complex than a simple glucosuria model. In a seven-patient sequencing study, pyuria during SGLT2-inhibitor treatment was associated with markedly reduced urinary microbial diversity and near-complete dominance by *Escherichia–Shigella* [42]. However, larger clinical studies did not consistently identify increased bacterial UTI risk. A propensity-matched claims analysis found a lower UTI risk but a higher genital bacterial infection risk with SGLT2 inhibitors than with dipeptidyl peptidase-4 inhibitors [43], while a cross-sectional clinical study also found no clear independent increase in UTI attributable to SGLT2 therapy [44]. Pyuria, asymptomatic bacteriuria, genital infection, and symptomatic bacterial UTI should therefore remain analytically distinct.

Observational cohorts reinforce the importance of metabolic severity rather than any single medication. Among hospitalized patients with type 2 diabetes, UTI was associated with older age, female sex, longer diabetes duration, higher BMI, and higher HbA1c [45]. Diabetes was also independently associated with recurrence in a large integrated health-system cohort [5]. These studies lack microbiome measurements but establish the clinical relevance of the metabolic context in which microbial changes occur.

### 4.4. Obesity: Vaginal Dysbiosis, Inflammation, and Experimental Bladder Vulnerability

Human evidence links obesity to vaginal ecological differences, although it is not specific to postmenopausal rUTI. Women with obesity had a lower prevalence of *Lactobacillus*-dominant communities, greater representation of diverse anaerobes, and higher vaginal concentrations of multiple inflammatory cytokines than non-obese controls [23]. Bariatric surgery did not produce a consistent overall microbiome shift at six months, but women with greater weight loss were more likely to have a *Lactobacillus*-dominant profile. In a larger reproductive-age cohort, overweight or obesity was associated with higher alpha diversity and less frequent *Lactobacillus* dominance after matching for demographic factors [24]. A small study additionally reported reduced urinary *Bifidobacterium* in obesity, prediabetes, and diabetes, but its limited sample and targeted quantitative PCR approach restrict inference [46].

Experimental data provide a direct obesity–UTI mechanism. Diet-induced obesity increased UPEC susceptibility in male and female mice, altered urothelial transcription, and activated focal-adhesion kinase signalling. Focal-adhesion kinase overexpression enhanced UPEC invasion in primary human urothelial cells [22]. These findings identify a plausible pathway by which obesity may weaken bladder resistance, but clinical validation in postmenopausal women is absent. Therefore, obesity should be treated as the least clinically validated component of the proposed framework. Current evidence supports biological plausibility, but not a confirmed postmenopausal obesity–microbiome–rUTI causal pathway.

### 4.5. Proposed Integrated Susceptibility Framework

Taken together, the available evidence supports a proposed, evidence-informed susceptibility framework rather than a proven causal pathway. Estrogen deficiency may alter epithelial maturation, vaginal pH, glycogen availability, Lactobacillus dominance, and local inflammatory tone. Diabetes may contribute through altered urogenital microbial profiles, hyperglycaemia-associated impairment of antimicrobial peptide expression, and experimental evidence of disrupted urothelial defence. Obesity may be associated with vaginal dysbiosis and inflammation, while experimental models suggest that obesity-related bladder signalling changes could increase uropathogenic *Escherichia coli* invasion. Microbial reservoirs in the gut, rectum, vagina, urinary tract, and bladder wall may contribute to repeated exposure or persistence in selected patients.

However, these components have generally been studied separately. No included study simultaneously measured menopausal status, estrogen exposure, glycaemic control, adiposity, host immunity, multi-compartment microbiota, strain-level reservoirs, antimicrobial exposure, and prospectively confirmed recurrence within the same cohort. Therefore, the urogenital microbiome–metabolic interface should be interpreted as a biologically plausible framework that generates testable hypotheses, not as an established causal axis. The relative contribution of each component is likely to vary among individuals and clinical phenotypes.

## 5. Clinical Evidence and Translational Implications

### 5.1. Vaginal Estrogen

Vaginal estrogen has the most direct intervention evidence in postmenopausal rUTI. In a randomized trial comparing an estradiol ring or conjugated estrogen cream with placebo, fewer women receiving vaginal estrogen experienced UTI within six months in both intention-to-treat and per-protocol analyses [47]. An ultra-low-dose 0.005% estriol gel trial similarly reported fewer infections, improved vaginal pH, and favourable short-term tolerability over 24 weeks [32]. These findings support a preventive effect across different local formulations, although both trials had limited duration and the earlier trial enrolled a small sample.

The biological response to estrogen is multidimensional. Vaginal estrogen has been associated with reduced urinary inflammation and interleukin-6 [3], enrichment of urogenital *Lactobacillus* or *Bifidobacterium* and correlations with urinary estrogen metabolites [31], and substantial remodelling of vaginal microbial and metabolic profiles [34]. In the randomized secondary analysis, 80% of women receiving estradiol had *Lactobacillus*- or *Bifidobacterium*-dominated communities after 12 weeks, compared with 36% using moisturizer and 26% receiving placebo; estradiol also lowered median pH and altered more than half of the measured metabolites [34]. A prospective estriol study in women with vaginal atrophy and stress incontinence likewise found lower pH and reduced diversity after treatment [48].

Not all studies demonstrate restoration of urinary *Lactobacillus*. Local estrogen altered the urinary microbiome in one small cohort mainly through reduced *Finegoldia magna*, without a significant increase in lactobacilli [35]. This inconsistency may reflect differences in vaginal versus urinary sampling, formulation, baseline ecology, treatment duration, and taxonomic resolution. Clinical recurrence reduction is therefore better supported than any single microbiome-mediated mechanism. Vaginal estrogen should be understood as an intervention that modifies epithelial, biochemical, inflammatory, and microbial conditions rather than as a narrowly probiotic therapy.

### 5.2. Lactobacillus and Other Microbiome-Directed Strategies

Evidence for direct *Lactobacillus* supplementation is promising but preliminary. In a small study of postmenopausal women, *L. crispatus*-containing vaginal suppositories increased median vaginal *Lactobacillus* abundance from undetectable levels to 19% and were associated with a reduction in annual cystitis episodes from 6.3 to 2.4 [36]. However, the prevention group was not a conventional randomized placebo-controlled cohort, and its vaginal community remained closer to the recurrent-cystitis profile than to healthy controls.

A pilot randomized trial of *Lactobacillus*-containing feminine hygiene products reported microbiome and symptom changes in mixed pre- and postmenopausal participants, but the population and outcomes do not establish rUTI prevention [49]. Live vaginal *Lactobacillus* treatment also improved selected GSM symptoms over short follow-up in a microbiome study, without demonstrating durable reduction in culture-confirmed rUTI [13]. Product composition, viability, dosing, colonization capacity, and baseline community state differ substantially across studies.

Cranberry provides a useful caution against assuming that clinical benefit requires global microbiome remodelling. In women with rUTI, long-term cranberry consumption produced little change in overall gut taxonomy, diversity, functional pathways, or *E. coli* abundance, although one low-abundance *Flavonifractor* taxon differed between groups [50]. Collectively, these studies do not yet support routine use of probiotic or other microbiome-directed interventions for rUTI prevention; local estrogen remains the better-supported preventive intervention in postmenopausal women.

### 5.3. Microbiome-Aware Diagnosis and Refractory Disease

Standard urine culture has clinically important limitations in rUTI. In symptomatic women, expanded quantitative urine culture detected more species and uropathogens than standard culture, while voided samples generated more false-positive findings than catheterized specimens [2]. Sequencing studies similarly identified distinct communities and increased organism detection in culture-negative or recurrent cystitis [7,8,37]. These methods may reveal organisms overlooked by standard aerobic culture, but greater analytical sensitivity does not necessarily improve clinical specificity.

Each platform answers a different question. Culture confirms viable organisms and permits susceptibility testing; 16S sequencing profiles bacterial communities but may have limited species resolution; shotgun metagenomics provides strain, functional, and resistance-gene information; metabolomics measures the biochemical environment; and fluorescence in situ hybridization localizes bacteria within tissue [31,33,40]. In postmenopausal rUTI, paired metagenomic and metabolomic analysis identified distinct microbe–metabolite networks, a lipid signature associated with active infection, and deoxycholic acid as a potential indicator of recurrence [33]. These findings are hypothesis-generating and require external validation before diagnostic use.

Microbiome-informed approaches may be most relevant in selected refractory cases. Persistence of the same organism after electrofulguration and the association between bladder-wall bacterial burden and relapse suggest that routine urine culture may incompletely represent tissue-resident infection [39,40]. Even in this setting, invasive sampling and advanced sequencing remain research tools, and no validated microbial abundance, diversity, metabolite, or resistance-gene threshold currently distinguishes colonization from disease.

### 5.4. Metabolic and Antimicrobial Considerations

Metabolic evaluation should accompany, rather than replace, established rUTI assessment. Diabetes, higher HbA1c, elevated BMI, and longer disease duration are associated with UTI or recurrence in large cohorts [5,46]. Mechanistic evidence supports attention to glycaemic control because hyperglycaemia impairs urothelial antimicrobial defence [18], but no included trial demonstrates that improving HbA1c prevents rUTI through microbiome restoration. Similarly, obesity management is biologically rational, yet weight loss has not been tested as a postmenopausal rUTI intervention with microbiome and recurrence endpoints.

SGLT2-inhibitor studies illustrate the need for precise outcome definitions. Small microbiome data link pyuria with marked urinary dysbiosis [42], whereas larger cohorts do not consistently show increased bacterial UTI and may show greater risk primarily for genital infection [43,44]. Medication decisions should therefore not be based on microbiome findings alone.

Finally, antimicrobial stewardship is central to this framework. Resistant organisms and resistance genes are enriched in recurrent disease, while repeated empirical treatment may further disrupt colonization resistance [4,31]. The available evidence supports accurate diagnosis, culture-guided treatment when feasible, and avoidance of unnecessary antibiotics. It does not yet support replacing standard care with sequencing-guided eradication of all detected organisms. The intervention evidence is summarized in Table 2.

## 6. Critical Synthesis and Clinical Positioning

### 6.1. Findings Supported Most Strongly

The most consistent evidence concerns menopause-associated ecological remodelling and local estrogen. Across urinary and vaginal studies, postmenopausal status is associated with reduced *Lactobacillus* dominance, higher pH, and greater representation of anaerobic or opportunistic communities [6,11,25,27]. These changes are not universal and do not map directly onto symptoms, but they establish menopause as a major determinant of urogenital ecology. Randomized and prospective studies further show that vaginal estrogen can reduce recurrence and modify pH, inflammatory activity, microbial composition, or metabolism [3,32,34,47]. The clinical reduction in rUTI is more firmly supported than any single microbiome-mediated mechanism.

Recurrent disease also cannot be explained solely by acute bladder infection. Longitudinal, multi-compartment, and tissue-based studies support contributions from gut, rectal, vaginal, urinary, and bladder-wall reservoirs [14,15,17,39]. Evidence is strongest for persistent exposure and tissue bacterial burden in selected refractory cases, but less consistent for a universal gut dysbiosis signature. Standard culture therefore captures only part of the biological context, although it remains essential for confirming viable organisms and antimicrobial susceptibility.

### 6.2. Findings Supported Moderately

Diabetes is supported as a clinically relevant modifier through converging microbial, epidemiological, and mechanistic evidence. Studies report altered urinary or vaginal communities, including reduced *Lactobacillus*, increased bacterial burden, and associations with glycaemic indices [19,20,41,51]. Experimental work shows that hyperglycaemia and impaired insulin-receptor signalling weaken antimicrobial peptide expression and urothelial barrier integrity [18,21]. Direct prospective evidence that these pathways mediate postmenopausal rUTI remains unavailable.

The gut–bladder relationship is also supported moderately. Reduced gut richness, depletion of butyrate-producing taxa, altered immune profiles, and distinct *E. coli* transcriptional states have been described in women with rUTI [17,52]. Yet larger and older cohorts have not consistently shown that overall gut composition independently predicts recurrence [16,53]. Strain behaviour and host response may therefore be more important than broad community diversity.

### 6.3. Findings Supported Mainly by Indirect or Experimental Evidence

Obesity is the least directly established component. Human studies associate obesity with reduced vaginal *Lactobacillus* dominance, increased diversity, and local inflammation, but these cohorts were predominantly reproductive-aged or not designed around postmenopausal rUTI [23,24]. Additional metabolic and menopause-focused studies cannot establish an obesity-driven recurrence pathway [46,54]. Experimental evidence that obesity activates bladder focal-adhesion signalling and increases UPEC invasion is biologically important, but clinical confirmation is lacking [22].

Likewise, available data do not establish that SGLT2 inhibitor-associated microbiome changes cause bacterial UTI. Small sequencing studies link pyuria with dysbiosis, whereas larger analyses show no consistent increase in bacterial UTI and distinguish this outcome from genital infection [42,43,44]. The directness and strength of evidence across the proposed interface are summarized in Table 3.

### 6.4. Anatomical, Neurological, and Functional Modifiers in Older Women

The comparatively short female urethra provides a relatively short route for ascending microbial exposure from the vaginal, periurethral, and perineal regions. In a three-dimensional transvaginal ultrasound study of women undergoing minimally invasive sacrocolpopexy, the mean urethral length was approximately 30 mm and did not change significantly after prolapse repair [55]. In postmenopausal women, this anatomical susceptibility coexists with vulvovaginal and periurethral atrophy, reduced estrogen-dependent epithelial support, higher vaginal pH, and altered local microbial ecology. These features may facilitate colonization or ascending exposure but do not independently establish symptomatic infection.

Pelvic organ prolapse may contribute to UTI susceptibility principally when it is accompanied by ineffective bladder emptying, increased postvoid residual urine, or catheter exposure. In women undergoing surgery for pelvic organ prolapse, advanced prolapse was independently associated with postoperative voiding dysfunction, which increased catheter burden and the potential for catheter-associated UTI [56]. A separate urodynamic cohort demonstrated that more advanced apical prolapse was associated with voiding symptoms and a postvoid residual volume exceeding 100 mL [57]. These findings do not establish pelvic organ prolapse as an independent universal cause of rUTI; rather, they support evaluating prolapse together with voiding symptoms, postvoid residual urine, and catheter requirements.

Age-related changes in bladder function may further impair urinary clearance. In a cohort of 602 neurologically intact women with voiding dysfunction but without cystocele, the prevalence of detrusor underactivity increased with advancing age [58]. Experimental evidence also suggests a possible structural mechanism: in an ovariectomized mouse model of estrogen deficiency, increased urinary frequency was accompanied by bladder-tissue remodelling and greater collagen III deposition, while estradiol treatment reduced collagen accumulation and improved bladder function [59]. These findings provide mechanistic support for an association among estrogen depletion, collagen remodelling, and lower-urinary-tract dysfunction. However, the animal findings do not prove that collagen deposition directly causes detrusor underactivity or rUTI in older postmenopausal women.

Stroke, immobility, functional disability, urinary retention, and catheter exposure represent additional clinically relevant modifiers. In a large multicentre cohort of immobile patients with stroke, UTI risk was associated with increasing age, female sex, prolonged hospitalization, and longer catheterization duration [60]. Among patients undergoing postacute stroke rehabilitation, those who developed UTI had lower functional-independence and ambulation scores, while admission with a urinary catheter independently predicted an earlier UTI event [61]. A separate cohort of patients with acute ischaemic stroke reported urinary retention in approximately 11% of patients; severe functional dependence, female sex, and UTI were independently associated with retention [62]. Together, these findings indicate that stroke-related neurological dysfunction, limited mobility, ineffective emptying, and catheter exposure may interact to increase UTI susceptibility. In bedridden or highly dependent women, incontinence and dependence on others for toileting and hygiene may also plausibly increase perineal microbial exposure, although perineal contamination was not directly quantified in these studies. Bacteriuria should remain distinguished from symptomatic UTI and microbiologically confirmed recurrent infection.

**Figure 3:**
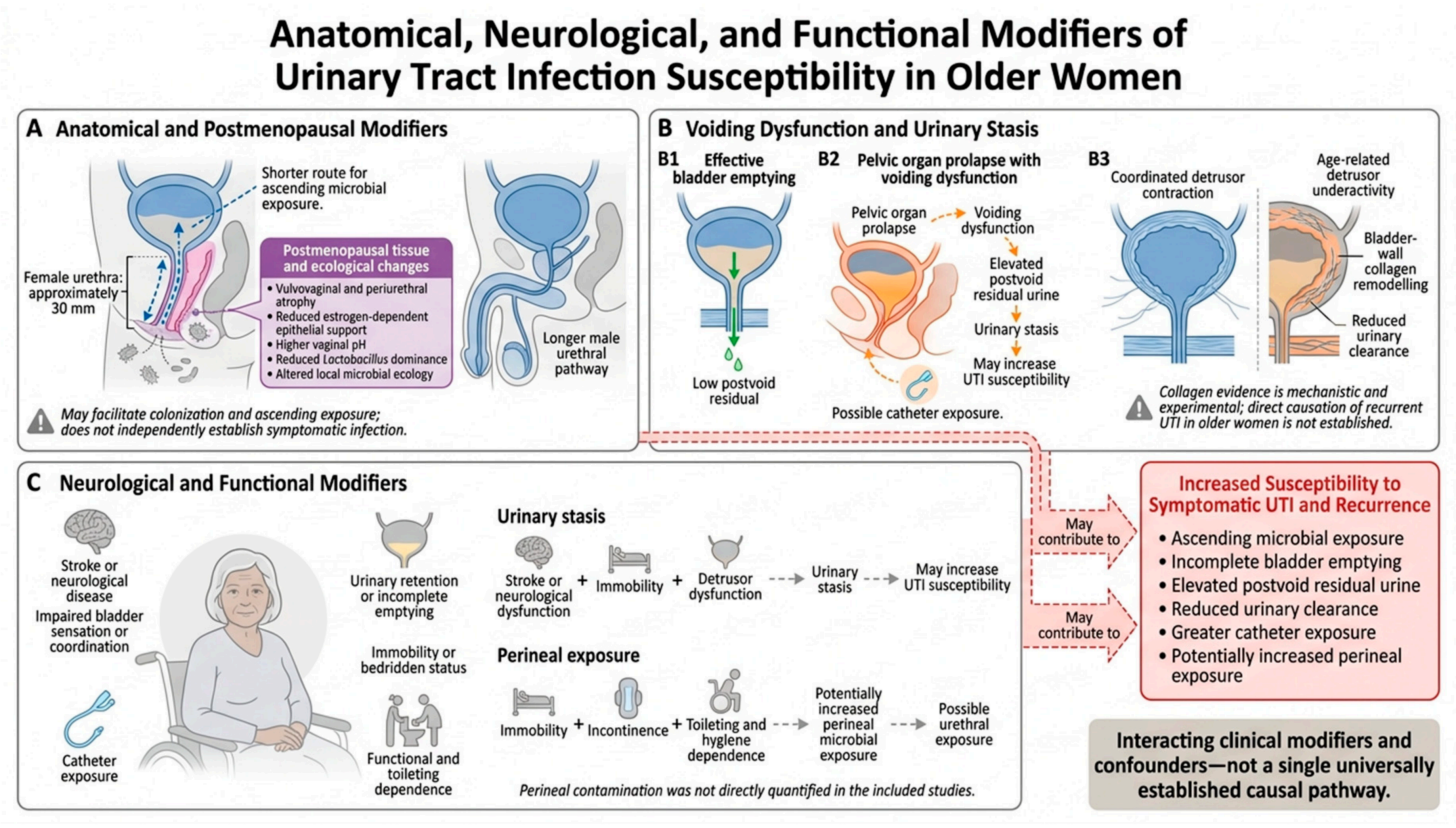
Anatomical, neurological, and functional modifiers of urinary tract infection susceptibility in older women. The short female urethra, postmenopausal tissue changes, pelvic organ prolapse with impaired emptying, detrusor dysfunction, stroke, immobility, functional dependence, incontinence, urinary retention, and catheter exposure may increase susceptibility through ascending microbial exposure, urinary stasis, or reduced urinary clearance. These factors are interacting clinical modifiers rather than a single established causal pathway. Abbreviations: PVR, postvoid residual; rUTI, recurrent urinary tract infection; UTI, urinary tract infection. Created in BioRender. Alabid, I. (2026) https://BioRender.com/tix59pi.

Overall, anatomical and functional assessment should complement hormonal, microbial, and metabolic evaluation in older women with rUTI. Clinically relevant factors include pelvic organ prolapse, voiding symptoms, postvoid residual urine, detrusor function, previous stroke or other neurological disease, mobility and functional status, incontinence, toileting dependence, bedridden status, and catheter exposure. These variables are potential susceptibility modifiers and confounders rather than components of a single established causal pathway. The anatomical, neurological, and functional modifiers discussed above are summarized in Figure 3.

### 6.5. Clinical Positioning

Current evidence supports phenotype-aware assessment rather than a microbiome-based treatment algorithm. Vaginal estrogen has the clearest preventive evidence in hypoestrogenic women. Diabetes control, obesity management, bladder-function assessment, and antimicrobial stewardship remain clinically rational, but their microbiome-mediated benefits are unproven. Enhanced culture, sequencing, metabolomics, and tissue analysis may clarify selected refractory cases, yet no validated microbial threshold distinguishes colonization from disease or guides routine therapy [6,33,37,40]. The proposed interface should therefore be viewed as an evidence-informed research framework, not a validated diagnostic or causal model.

## 7. Future Directions

### 7.1. Longitudinal, Multi-Compartment Cohorts

The principal research priority is prospective sampling across the entire recurrence cycle. Studies should obtain catheterized urine, vaginal samples, rectal swabs, stool, blood, hormonal measurements, HbA1c, and adiposity data during asymptomatic periods, acute infection, treatment, and recovery. Simultaneous sampling is necessary to distinguish persistent reservoirs from newly acquired organisms and to determine whether microbial changes precede recurrence or result from infection and antibiotics [14,15,16,17].

### 7.2. Methodological Standardization

Future studies should use consistent rUTI definitions, standardized specimen collection, appropriate negative and environmental controls, and absolute microbial quantification. Reporting should include recent antibiotics, local or systemic estrogen, probiotics, sexual activity, incontinence, postvoid residual volume, diabetes treatment, and SGLT2-inhibitor exposure. Harmonization of culture, full-length 16S sequencing, shotgun metagenomics, and metabolomics is needed to improve comparability across studies [2,9,37,54].

### 7.3. Causal and Strain-Level Investigation

Strain-resolved approaches should track uropathogens across the gut, rectum, vagina, urine, and bladder tissue. These studies should test whether recurrence reflects repeated ascent, intracellular or bladder-wall persistence, or both. Functional analyses should also examine microbial transcription, resistance genes, and host–microbe metabolite interactions rather than relying on relative taxonomic abundance alone [33,39,40,52].

### 7.4. Biomarker Development

Candidate biomarkers require external validation in independent postmenopausal cohorts. Future prediction models should integrate microbial taxa, resistance genes, metabolites, estrogen status, inflammatory markers, HbA1c, BMI, and prior recurrence rather than evaluating each factor separately. Promising signals, including recurrence-associated metabolites and diabetes-related vaginal community changes, should be assessed for discrimination, calibration, and clinical utility [19,31,33,41].

### 7.5. Intervention Trials

Intervention studies should incorporate both clinical recurrence and standardized microbiome endpoints. Priorities include comparisons of estrogen formulations, estrogen combined with vaginal *Lactobacillus*, metabolic optimization, weight-loss interventions in postmenopausal women, antibiotic-sparing strategies, and reservoir-targeted approaches for refractory disease [32,36,47,49]. Trials should be sufficiently long to assess durable recurrence, antimicrobial exposure, resistance selection, and whether ecological changes mediate clinical benefit.

## 8. Limitations of the Current Evidence

The present evidence base is constrained by substantial methodological and clinical heterogeneity. Many microbiome studies enrolled fewer than 100 participants, used cross-sectional designs, and differed in definitions of recurrent UTI, menopausal status, symptom status, and recent antimicrobial exposure. Consequently, temporal direction is often uncertain: dysbiosis may precede infection, result from infection, or reflect prior treatment. Differences in participant age, estrogen use, diabetes severity, obesity, incontinence, sexual activity, and bladder emptying further limit direct comparison across cohorts [9,10,19,28].

Sampling and analytical methods introduce additional uncertainty. Catheterized and voided urine capture different anatomical signals, and voided specimens may contain urethral or vulvovaginal organisms. Standard culture, expanded culture, 16S rRNA sequencing, shotgun metagenomics, metabolomics, and fluorescence in situ hybridization measure different biological features and are not interchangeable [2,30,37,40]. Low microbial biomass increases susceptibility to contamination, while sequencing detects DNA without proving viability. Relative-abundance analyses may also obscure changes in absolute microbial load.

Additional microbiome-specific limitations should be considered. Low-biomass urinary samples are susceptible to reagent, environmental, and procedural contamination, and not all studies used comparable negative controls or contamination-filtering approaches. Differences in DNA extraction, primer choice, sequencing depth, reference databases, and bioinformatic pipelines may affect taxonomic assignment and comparability across studies. Most 16S rRNA studies provide limited species- or strain-level resolution, whereas shotgun metagenomics may better characterize functional potential but still does not prove organism viability or host invasion. Finally, compositional relative-abundance analyses may obscure absolute changes in microbial burden, and batch effects may introduce apparent differences unrelated to biology. These limitations reduce confidence in any single microbial signature and support the need for standardized, multi-compartment, longitudinal studies.

The evidence is uneven across the proposed interface. Direct human evidence is strongest for menopause-associated ecological change and vaginal estrogen, whereas diabetes is supported by a combination of clinical, microbiome, and mechanistic studies. The obesity component relies largely on reproductive-age cohorts and experimental models, limiting extrapolation to postmenopausal rUTI [22,23,24,46].

Finally, few studies integrate hormonal status, glycaemic control, adiposity, host immunity, microbial function, and prospective recurrence within the same cohort. No validated microbial, metabolomic, or inflammatory signature currently has sufficient reproducibility or clinical utility to guide routine diagnosis or treatment.

## 9. Conclusions

Postmenopausal recurrent urinary tract infection is best understood as a multifactorial disorder rather than the consequence of estrogen deficiency or pathogen exposure alone. Current evidence supports interactions among hormonal remodelling, altered urogenital ecology, impaired urothelial defence, metabolic dysfunction, microbial reservoirs, and antimicrobial pressure. The evidence is strongest for menopause-associated microbial change and vaginal estrogen therapy, moderate for diabetes-related susceptibility and extra-urinary reservoirs, and limited for a direct obesity–microbiome–rUTI pathway.

The proposed urogenital microbiome–metabolic interface is therefore a useful evidence-based framework, but it is not yet a validated causal or diagnostic model. Standard clinical evaluation, appropriate urine culture, local estrogen in suitable patients, metabolic assessment, and antimicrobial stewardship remain the practical foundation of care. Microbiome profiling, metabolomic biomarkers, and reservoir-targeted therapies remain investigational. Progress will require longitudinal, multi-compartment studies that integrate hormonal status, metabolic health, host immunity, microbial function, and clinically confirmed recurrence.

## Figures and Tables

**Table 1:** Characteristics and principal findings of ten representative studies included in the review. Abbreviations: AMR, antimicrobial resistance; BMI, body mass index; FAK, focal adhesion kinase; GSM, genitourinary syndrome of menopause; LC–MS/MS, liquid chromatography–tandem mass spectrometry; PM, postmenopausal; rUTI, recurrent urinary tract infection; T2D, type 2 diabetes; UPEC, uropathogenic *Escherichia coli*; UTI, urinary tract infection.

Reference	Country and Study Design	Population and Sample Size	Exposure or Comparison	Specimens and Analytical Methods	Principal Findings	Main Limitations
Ammitzbøll et al., 2021 [6]	Denmark; cross-sectional comparative study	54 asymptomatic women analyzed: 34 premenopausal and 20 postmenopausal	Premenopausal versus postmenopausal status	Catheterized urine; 16S rRNA sequencing	Postmenopause was associated with higher urinary diversity, lower *Lactobacillus*, and greater *Gardnerella*/*Prevotella* abundance.	Low-biomass exclusions; asymptomatic cohort; rUTI not assessed.
Neugent et al., 2022 [31]	United States; controlled cross-sectional cohort	75 postmenopausal women: no UTI history, inactive rUTI history, or active UTI	rUTI history, active UTI, and estrogen therapy	Urine; shotgun metagenomics, advanced culture, estrogen metabolites, functional and AMR-gene analyses	rUTI and active UTI were linked to taxonomic and functional shifts; estrogen exposure correlated with *Lactobacillus*/*Bifidobacterium*; AMR genes were enriched with rUTI history.	Cross-sectional design; active-UTI group was older; exploratory associations require validation.
Muiños Fernández et al., 2024 [32]	Spain; multicentre randomized double-blind placebo-controlled trial	108 postmenopausal women with GSM and rUTI	Ultra-low-dose 0.005% estriol vaginal gel versus placebo moisturizing gel	Clinical UTI surveillance, antibiotic use, vaginal pH, safety, and tolerability over 24 weeks	Estriol reduced UTI incidence and improved vaginal pH over follow-up.	24-week follow-up; no sequencing or direct microbiome endpoints.
Neugent et al., 2025 [33]	United States; secondary multi-omic analysis	Same 75-woman postmenopausal cohort as Neugent et al. [31]	UTI history and active infection status	Paired urinary shotgun metagenomics and targeted LC–MS/MS metabolomics	Microbe–metabolite networks differed; a urinary lipid signature marked active UTI; deoxycholic acid was linked to shorter recurrence-free time.	Secondary analysis; cross-sectional metabolomics; biomarkers not externally validated.
Horseman et al., 2026 [15]	United States; cross-sectional case–control study	62 postmenopausal women: 31 with rUTI and 31 controls	rUTI versus no rUTI	Vaginal 16S sequencing, urinary pathogen/AMR panel, rectal culture, and historical urine cultures	rUTI was associated with vaginal dysbiosis, higher AMR burden, resistant rectal uropathogens, and cross-compartment concordance.	Cross-sectional design; historical urine cultures were not simultaneous; modest single-centre sample.
Worby et al., 2022 [17]	United States; prospective one-year longitudinal multi-omic cohort	31 women: 15 with rUTI history and 16 controls; 24 UTI episodes	rUTI history and longitudinal infection status	Monthly stool samples; urine, stool, and blood at enrolment/UTI visits; shotgun metagenomics and transcriptomics	rUTI history was linked to lower gut richness, fewer butyrate-producing taxa, and immune differences; total gut *E. coli* was broadly similar.	Small cohort; not postmenopause-specific; antibiotic exposure and interindividual variation limited causal inference.
Mohanty et al., 2022 [18]	Sweden; translational human, animal, and cellular study	Human diabetes/control samples plus clamp studies, diabetic mice, and uroepithelial cells	Diabetes, glucose, insulin, and estradiol exposure	Urine cells/proteins, clamp studies, mouse models, uroepithelial cultures, gene/protein assays, and experimental *E. coli* infection	High glucose reduced psoriasin, impaired epithelial barrier function, and increased *E. coli* burden; estradiol improved bacterial killing in cells.	Heterogeneous experimental components; not selected for rUTI or postmenopause; several findings were model-based.
Qiu et al., 2025 [19]	China; cross-sectional metagenomic case–control study	45 peri- or postmenopausal women: 22 with T2D and 23 controls	T2D versus no diabetes	Vaginal shotgun metagenomics, diversity analysis, differential abundance, and network modelling	T2D was associated with higher diversity, lower *Lactobacillus*, enrichment of opportunistic taxa, and glycaemic correlations.	Small sample; cross-sectional design; no prospective UTI/rUTI endpoint.
Raglan et al., 2021 [23]	United Kingdom; case–control study with bariatric-surgery subset	109 women: 67 with obesity and 42 without obesity; 27 followed after bariatric surgery	Obesity versus non-obesity and change after bariatric surgery	Vaginal 16S sequencing, cytokine assays, and 6-month follow-up	Obesity was linked to lower *Lactobacillus* dominance, higher diversity, anaerobe enrichment, and local inflammation; greater weight loss correlated with *Lactobacillus* dominance.	Not postmenopause- or rUTI-specific; residual obesity after surgery; observational design.
Schwartz et al., 2025 [22]	United States; translational animal and cellular study	High-fat-diet mouse model with complementary human urothelial-cell experiments	Diet-induced obesity and FAK manipulation	UPEC infection model, bladder transcriptomics, protein assays, immunostaining, and FAK overexpression	Obesity increased UPEC susceptibility and activated FAK/extracellular-matrix signalling; FAK overexpression promoted urothelial invasion.	Predominantly experimental; no postmenopausal clinical cohort; mouse obesity may not fully represent human rUTI.

**Table 2:** Clinical, microbiome-related, and refractory-disease interventions relevant to postmenopausal recurrent urinary tract infection. Abbreviations: GSM, genitourinary syndrome of menopause; rUTI, recurrent urinary tract infection; UTI, urinary tract infection.

Intervention/Study	Population and Design	Principal Endpoint	Main Finding	Key Limitation
Vaginal estrogen ring or cream [47]	Postmenopausal women with rUTI; randomized trial	UTI recurrence over 6 months	Fewer recurrences occurred with vaginal estrogen than with placebo.	Small sample; short follow-up; microbiome not primary endpoint.
Vaginal estradiol [34]	Postmenopausal women; secondary randomized-trial analysis	Vaginal microbiota, pH, and metabolome	Lower pH, greater *Lactobacillus*/*Bifidobacterium* dominance, and broad metabolomic shifts.	Secondary analysis; clinical rUTI recurrence not primary endpoint.
Local estrogen and urinary microbiome [35]	Postmenopausal women with and without rUTI; prospective cohort	Urinary community composition	Reduced *Finegoldia magna* without consistent urinary *Lactobacillus* increase.	Small cohort; site-specific urinary microbiome response.
Ultra-low-dose estriol gel [32]	Postmenopausal women with GSM; randomized placebo-controlled trial	UTI prevention, pH, and tolerability	Fewer infections and improved vaginal pH over 24 weeks.	Limited duration; formulation-specific findings.
Vaginal estriol cream [48]	Postmenopausal women with stress incontinence; prospective study	Vaginal microbiota and pH	Lower pH and reduced microbial diversity after treatment.	Not designed around rUTI prevention.
*Lactobacillus crispatus* suppository [36]	Postmenopausal women with recurrent cystitis; small intervention cohort	Vaginal *Lactobacillus* and annual cystitis episodes	Increased *Lactobacillus* abundance and fewer reported cystitis episodes.	Limited sample; nonstandard comparator.
Lactobacillus-containing hygiene products [49]	Pre- and postmenopausal women; pilot randomized trial	Symptoms and vaginal microbiome	Short-term symptom and microbiome changes were reported.	Mixed population; no validated rUTI endpoint.
Cranberry [50]	Women with rUTI; placebo-controlled microbiome analysis	Gut microbiome composition	Minimal global gut microbiome change despite intervention.	Microbiome was a secondary outcome; limited mechanistic inference.
Electrofulguration [39,40]	Postmenopausal women with antibiotic-recalcitrant rUTI	Recurrence and bladder-wall bacterial persistence	Persistent organisms and higher tissue bacterial burden were associated with relapse in selected patients.	Invasive, nonrandomized, and applicable mainly to refractory disease.

**Table 3:** Evidence hierarchy for major conclusions in the proposed urogenital microbiome–metabolic framework.

Major Conclusion	Main Supporting Evidence	Evidence Category	Interpretation
Vaginal estrogen reduces rUTI recurrence in postmenopausal women.	Randomized and prospective clinical intervention studies [3,32,47]	High-to-moderate clinical evidence	Recurrence benefit is supported; the exact microbiome-mediated mechanism remains incomplete.
Menopause is associated with altered vaginal and urinary microbial ecology.	Cross-sectional and longitudinal human microbiome studies [6,10,11,12]	Moderate human observational evidence	Menopause is a consistent ecological modifier, but no universal postmenopausal microbiome exists.
rUTI is associated with altered urinary or vaginal microbial profiles.	Human urinary/vaginal microbiome studies [7,8,36,37]	Low-to-moderate observational evidence	Broad associations are reported, but no validated diagnostic microbial signature exists.
Gut, rectal, vaginal, and bladder-wall reservoirs may contribute to recurrence.	Longitudinal, multi-compartment, culture, sequencing, and tissue-based studies [14,15,17,39]	Moderate mechanistic-clinical evidence	Reservoirs are plausible and clinically relevant in selected patients; transmission direction remains uncertain.
Bladder-wall persistence may contribute to refractory recurrence.	Tissue-based and electrofulguration studies [39,40]	Low-to-moderate evidence in selected refractory disease	Relevant mainly to antibiotic-recalcitrant cases; not generalizable to all postmenopausal rUTI.
Diabetes is associated with altered urogenital microbiota and UTI risk.	Human microbiome studies and clinical risk-factor cohorts [5,19,20,41]	Moderate observational evidence	Diabetes is a clinically relevant modifier; microbiome-mediated recurrence remains unproven.
Hyperglycaemia and impaired insulin signalling may weaken urothelial defence.	Human-cell, animal, and translational mechanistic studies [18,21]	Translational/hypothesis-generating evidence	Biologically plausible, but not proven in postmenopausal rUTI.
SGLT2-inhibitor-associated microbiome changes and bacterial UTI risk remain uncertain.	Small sequencing study and larger clinical cohorts [42,43,44]	Low/mixed evidence	Pyuria, bacteriuria, genital infection, and symptomatic bacterial UTI must be separated.
Obesity is associated with vaginal dysbiosis and inflammation.	Human obesity and vaginal microbiome studies [23,24]	Low direct evidence for postmenopausal rUTI	Relevant to the framework, but most evidence is not specific to postmenopausal rUTI.
Obesity may increase bladder susceptibility to UPEC.	Diet-induced obesity and urothelial-cell experiments [22]	Experimental/hypothesis-generating evidence	Biologically plausible, but not clinically validated in postmenopausal women.

## Data Availability

No new data were created or analyzed in this study. Data sharing is not applicable to this article.

## Supplementary Materials

The following supporting information can be downloaded at: https://www.mdpi.com/article/10.3390/jcm15155895/s1, File S1: Complete database-specific PubMed and Scopus search strategies.

## Abbreviations

Abbreviation	Full term
AMR	Antimicrobial resistance
BMI	Body mass index
DNA	Deoxyribonucleic acid
*E. coli*	*Escherichia coli*
FAK	Focal adhesion kinase
FISH	Fluorescence in situ hybridization
GSM	Genitourinary syndrome of menopause
HbA1c	Glycated hemoglobin
IL-6	Interleukin-6
LC–MS/MS	Liquid chromatography–tandem mass spectrometry
NF-κB	Nuclear factor kappa B
pH	Potential of hydrogen
qPCR	Quantitative polymerase chain reaction
rRNA	Ribosomal ribonucleic acid
rUTI	Recurrent urinary tract infection
SGLT2	Sodium–glucose cotransporter 2
UPEC	Uropathogenic *Escherichia coli*
UTI	Urinary tract infection
16S rRNA	16S ribosomal ribonucleic acid
