# Supplementary material for: The Urogenital Microbiome–Metabolic Interface in Postmenopausal Recurrent Urinary Tract Infection: Estrogen Deficiency, Diabetes, Obesity, and Microbial Reservoirs
Source: J Clin Med. 2026 Jul 28;15(15):5895. doi: 10.3390/jcm15155895 (PMC13467049; doi:10.3390/jcm15155895)
Supplement: Supplementary file 1 [file jcm-15-05895-s001.zip › jcm-4432534-supplementary.pdf]

Supplementary File S1. Complete database-specific PubMed and Scopus search strategies

This supplementary file provides the complete PubMed and Scopus search strings used for the structured narrative review.

Scopus:

(TITLE-ABS-KEY ((({recurrent urinary tract infection} OR {recurrent UTI} OR {recurrent cystitis}) AND (women OR woman OR female OR postmenopaus* OR menopause) AND (microbiom* OR microbiota OR urobiome OR dysbiosis OR lactobacillus OR estrogen OR estradiol OR diabetes OR hyperglyc* OR obesity OR reservoir* OR resistome OR {antimicrobial resistance} OR {bladder wall})) OR ((postmenopaus* OR menopause) AND ({vaginal microbiome} OR {vaginal microbiota} OR {urinary microbiome} OR {urinary microbiota} OR {urogenital microbiome} OR urobiome))) OR TITLE (({urethral length} AND sacrocolpopexy) OR ({postoperative voiding dysfunction} AND {pelvic organ prolapse}) OR ({vaginal wall prolapse} AND {voiding dysfunction}) OR ({detrusor underactivity} AND {female voiding dysfunction}) OR (estrogen AND {bladder structure} AND voiding) OR ({post-stroke urinary tract infection} AND immobile) OR ({postacute stroke} AND {urinary tract infections}) OR ({urinary retention} AND {acute ischemic stroke}))) AND PUBYEAR > 2020 AND PUBYEAR < 2027 AND DOCTYPE (ar) AND LANGUAGE (English)

Pubmed:

((("recurrent urinary tract infection"[Title/Abstract] OR "recurrent urinary tract infections"[Title/Abstract] OR "recurrent UTI"[Title/Abstract] OR rUTI[Title/Abstract] OR "recurrent cystitis"[Title/Abstract] OR ((recurrent[Title/Abstract] OR recurrence[Title/Abstract]) AND ("urinary tract infection"[Title/Abstract] OR "urinary tract infections"[Title/Abstract] OR UTI[Title/Abstract] OR cystitis[Title/Abstract]))) AND (women[Title/Abstract] OR woman[Title/Abstract] OR female[Title/Abstract] OR postmenopaus*[Title/Abstract] OR menopause[Title/Abstract] OR menopaus*[Title/Abstract] OR perimenopaus*[Title/Abstract] OR "genitourinary syndrome of menopause"[Title/Abstract] OR GSM[Title/Abstract]) AND (microbiome[Title/Abstract] OR microbiota[Title/Abstract] OR urobiome[Title/Abstract] OR "urinary microbiome"[Title/Abstract] OR "vaginal microbiome"[Title/Abstract] OR "urogenital microbiome"[Title/Abstract] OR "gut microbiome"[Title/Abstract] OR dysbiosis[Title/Abstract] OR Lactobacillus[Title/Abstract] OR metagenomic*[Title/Abstract] OR

metabolomic*[Title/Abstract] OR "16S rRNA"[Title/Abstract] OR reservoir*[Title/Abstract] OR rectal[Title/Abstract] OR intestinal[Title/Abstract] OR "bladder wall"[Title/Abstract] OR intracellular[Title/Abstract] OR "antimicrobial resistance"[Title/Abstract] OR resistome[Title/Abstract] OR "expanded quantitative urine culture"[Title/Abstract] OR EQUC[Title/Abstract] OR estrogen[Title/Abstract] OR oestrogen[Title/Abstract] OR estradiol[Title/Abstract] OR estriol[Title/Abstract] OR diabetes[Title/Abstract] OR "type 2 diabetes"[Title/Abstract] OR hyperglyc*[Title/Abstract] OR SGLT2[Title/Abstract] OR obesity[Title/Abstract] OR obese[Title/Abstract] OR adiposity[Title/Abstract] OR "body mass index"[Title/Abstract] OR BMI[Title/Abstract] OR antibiotic*[Title/Abstract] OR antimicrobial*[Title/Abstract])) OR ("urethral length"[Title] AND (sacrocolpopexy[Title/Abstract] OR "pelvic organ prolapse"[Title/Abstract])) OR ("postoperative voiding dysfunction"[Title] AND "pelvic organ prolapse"[Title/Abstract]) OR ("vaginal wall prolapse"[Title] AND "voiding dysfunction"[Title/Abstract]) OR ("detrusor underactivity"[Title] AND (female[Title/Abstract] OR women[Title/Abstract] OR "female voiding dysfunction"[Title/Abstract])) OR (estrogen[Title] AND ("bladder structure"[Title/Abstract] OR "bladder remodelling"[Title/Abstract] OR "bladder remodeling"[Title/Abstract] OR collagen[Title/Abstract]) AND (voiding[Title/Abstract] OR "lower urinary tract symptoms"[Title/Abstract])) OR (("post-stroke"[Title] OR poststroke[Title]) AND ("urinary tract infection"[Title/Abstract] OR "urinary tract infections"[Title/Abstract] OR UTI[Title/Abstract]) AND (immobile[Title/Abstract] OR immobility[Title/Abstract] OR catheter*[Title/Abstract])) OR ("postacute stroke"[Title/Abstract] AND ("urinary tract infection"[Title/Abstract] OR "urinary tract infections"[Title/Abstract]) AND rehabilitation[Title/Abstract]) OR (("acute ischemic stroke"[Title] OR "acute ischaemic stroke"[Title]) AND "urinary retention"[Title/Abstract])) AND ("2021/01/01"[Date - Publication] : "2026/06/15"[Date - Publication]) AND English[Language] NOT (Review[Publication Type] OR Systematic Review[Publication Type] OR Meta-Analysis[Publication Type] OR Editorial[Publication Type] OR Letter[Publication Type] OR Comment[Publication Type] OR Protocol[Title])
